# Patterning Cu nanostructures tailored for CO_2_ reduction to electrooxidizable fuels and oxygen reduction in alkaline media†

**DOI:** 10.1039/c9na00166b

**Published:** 2019-05-20

**Authors:** Magdalena Michalak, Agata Roguska, Wojciech Nogala, Marcin Opallo

**Affiliations:** Institute of Physical Chemistry, Polish Academy of Sciences Kasprzaka 44/52 01-224 Warsaw Poland wnogala@ichf.edu.pl mopallo@ichf.edu.pl

## Abstract

Due to the limited availability of noble metal catalysts, such as platinum, palladium, or gold, their substitution by more abundant elements is highly advisable. Considerably challenging is the controlled and reproducible synthesis of stable non-noble metallic nanostructures with accessible active sites. Here, we report a method of preparation of bare (ligand-free) Cu nanostructures from polycrystalline metal in a controlled manner. This procedure relies on heterogeneous localized electrorefining of polycrystalline Cu on indium tin oxide (ITO) and glassy carbon as model supports using scanning electrochemical microscopy (SECM). The morphology of nanostructures and thus their catalytic properties are tunable by adjusting the electrorefining parameters, *i.e.*, the electrodeposition voltage, the translation rate of the metal source and the composition of the supporting electrolyte. The activity of the obtained materials towards the carbon dioxide reduction reaction (CO_2_RR), oxygen reduction reaction (ORR) in alkaline media and hydrogen evolution reaction (HER), is studied by feedback mode SECM. Spiky Cu nanostructures obtained at a high concentration of chloride ions exhibit enhanced electrocatalytic activity. Nanostructures deposited under high cathodic overpotentials possess a high surface-to-volume ratio with a large number of catalytic sites active towards the reversible CO_2_RR and ORR. The CO_2_RR yields easily electrooxidizable compounds – formic acid and carbon monoxide. The HER seems to occur efficiently at the crystallographic facets of Cu nanostructures electrodeposited under mild polarization.

## Abstract 2

Bare (non-capped) copper nanocatalysts were obtained from a polycrystalline metallic source.

## Graphical abstract

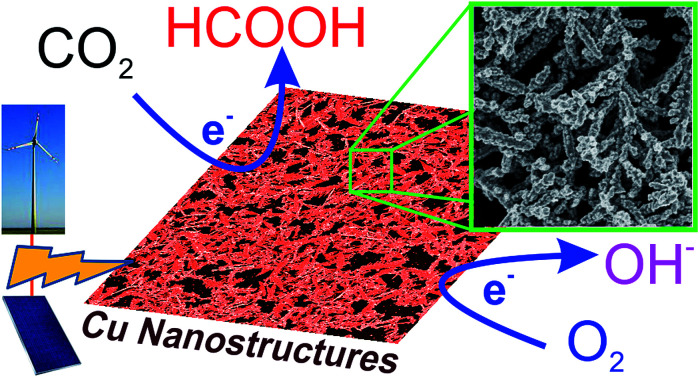

## Introduction

The unique properties of nanostructured materials attributed to their increased surface area, altered electronic states, or lattice structure are utilized in many applications. One of the most important technologies, which uses nanomaterials and affects society and the environment, is energy conversion and storage.^1^ The carbon dioxide reduction reaction (CO_2_RR) and oxygen reduction reaction (ORR) are the key chemical processes employed in these technologies. The crucial issue in the further development of these technologies is the tunable preparation of nanostructured electrode materials for better efficiency of electrode reactions.^2^ Deposition of nanoobjects on a conductive surface is one of the possibilities for tailoring the electrode structure.^3,4^ Apart from nanoobjects of various shapes, researchers focus on the formation of larger objects with a unique surface structure at the nanoscale and their catalytic properties.^5^ Such materials are commonly called nanostructures. Although nanostructured surfaces possess dimensions that are larger than those of individual nanoobjects (*e.g.* nanoparticles, nanotubes, and nanorods), they preserve features such as a large exposed surface to volume ratio and a large number of low coordination surface atoms (at edges, corners and vacancies).^6,7^ These features are usually beneficial for (electro)catalysis providing numerous catalytic centers to enhance the kinetics of heterogeneous processes.

Copper is the most abundant group 11 metallic element and one of the most abundant transition metals. Besides its use as a coinage metal, in many applications it is a cost efficient material competitive with noble metals (*e.g.* Au, Pt, and Pd). Plenty of potential applications of copper nanostructures (CuNSs) have already been proposed. Roughened copper is well known as an effective material for applications utilizing localized surface plasmon resonance (LSPR), such as surface enhanced Raman spectroscopy (SERS)^8,9^ and surface enhanced fluorescence.^10^ Due to the high thermal conductivity and volumetric heat capacity of copper, its nanostructures are used as dopants for thermal energy storage materials^11^ and thermal interface materials.^12^ The uniquely high absorbance of nanostructured Cu surfaces in a broad spectrum covering ultraviolet, visible and infrared radiation allows their use in photothermal conversion.^13^ They were also applied as quantum dots in solar cells.^14^ Cu exhibits catalytic,^15^ photocatalytic,^16^ antibacterial,^16^ superhydrophobic and self-cleaning properties.^17^ Due to their high electric conductivity and electrocatalytic properties, CuNSs are useful electrode materials, *e.g.* for the electroreduction of NO_3_^−^ and H_2_O_2_.^18^ Although CuNSs are unstable in acidic environments under anodic polarization or unbiased conditions,^19^ their anodic treatment in alkaline solutions causes the formation of CuO_*x*_ nanostructures exhibiting catalytic activity towards electrooxidation of various organic substances, such as l-tyrosine,^20^ glucose,^21,22^ hydrazine,^22^ and water.^23,24^

Even though there are a number of successful applications of noble metal (Pt and Pd) based nanocatalysts to electrochemical conversion of CO_2_,^25–27^ copper is the most promising catalyst for the CO_2_RR yielding valuable, high energy density products such as hydrocarbons,^28–31^ alcohols,^28,29,32^ formic acid and other carbonyls.^28,29,32–35^ It was demonstrated that the morphology and therefore the electrocatalytic properties of Cu nanostructures towards the CO_2_RR can be tuned by the addition of phosphate and the electrodeposition potential^35^ or appropriate selection of the Cu complex precursor.^34^ Copper-based materials also exhibit electrocatalytic properties towards the ORR^36^ and are considered as a replacement for platinum.^37,38^ A low overpotential of the ORR in alkaline media with Cu nanoparticles^39^ and nanoflowers^40^ was reported.

There are plenty of surfactant-free^8,9,13,16–18,41,42^ and surfactant-assisted^11,14,39,40,43–46^ methods to obtain CuNSs, such as sonoelectrochemical^43^ precipitation of Cu(ii) complex nanocrystals and their further calcination,^16^ plasma-induced decomposition of Cu complexes,^47^ homogeneous hydrothermal synthesis by reduction of Cu(ii) salts with hydrazine,^14,44^ reduction in solution with NaBH_4_,^39,45^ microwave assisted synthesis,^8^ disproportionation of CuCl,^11,46^ biogenic synthesis by using bacteria, fungi, and plant extracts,^48^ simple redox replacement by immersing of an iron plate in Cu(ii) salt solution,^42^ electroless deposition,^49^ and thermal annealing of Cu_2_O-doped glasses in a hydrogen atmosphere.^9^ Surfactant-assisted methods of synthesis yield nanostructures with a protection layer on their surfaces, preventing their further growth and aggregation. Although properly coordinated surface ligands promote catalysis on metal nanoparticles *via* steric interactions and electronic modifications,^50,51^ their presence may hinder the access of reactants to the metal surface and decrease the catalytic activity.^52^ Therefore bare (non-capped) nanostructures are desirable for catalysis.

For electrocatalysis the most useful are CuNSs deposited on conductive surfaces. This can be achieved by laser ablation of copper surfaces,^13,41^ plasma etching,^19^ dealloying,^30,31^ electrochemical polishing,^53^ thermal annealing,^54^ or electrodeposition.^10,18,20,22,43,55,56^ Gowthaman and John demonstrated that the applied substrate potential during Cu electrodeposition affects the geometry of the obtained deposit. They obtained cubic, spherical, dendritic and prickly CuNSs from the same solution.^22^

Local electroless deposition of copper has been done by the Schmuki group on AFM-produced nanosized scratches on Si(111) surfaces covered with an organic monolayer.^49^ The same group electrodeposited micropatterns of CuNSs on a similar surface modified with an electron beam.^57^ In a method based on scanning ion-conductance microscopy (SICM),^58–60^ a micropipette with a Cu salt solution and Cu anode inside was used as a source of copper. Two-dimensional Cu microcircuits were fabricated by lateral scanning over an indium-tin oxide (ITO) cathode in a Cu-free electrolyte.^58^ Müller *et al.* applied a similar approach with bipotentiostatic control of both electrodes.^60^ The Unwin group developed a SICM-based method for the fabrication of three-dimensional Cu structures with a dual-channel nanopipette, with one channel for metal precursor delivery and the second for maintaining a constant distance between the nanopipette and electrodeposited metal.^59^ Another micropipette-based method for local electrodeposition has been proposed by Staemmler *et al.*^61^ A capillary filled with Cu salt solution and equipped with auxiliary and reference electrodes was brought close to the substrate working electrode to ensure its contact with the pipette electrolyte. This technique, called scanning electrochemical cell microscopy (SECCM),^62^ was widely employed by the Unwin group, also for local electrodeposition of other metals.^63–67^ The same technique was also used for visualization of increased activity towards the CO_2_RR at the grain boundaries of a polycrystalline Au electrode.^68^ A very similar approach utilizing scanning meniscus confined electrodeposition has been applied for the preparation of nanoscale Cu connections,^69^ line arrays,^70^ and three-dimensional nanostructures.^71^

One of the most powerful methods for localized deposition of microarrays of metallic nanostructures as well as for the analysis of their electrocatalytic properties is scanning electrochemical microscopy (SECM).^72,73^ SECM was used for localized electrodeposition of Cu microstructures using its direct mode of operation.^74^ In this mode a microelectrode (SECM tip) made of an inert metal (Pt) was used as the positionable auxiliary electrode. The source of copper was an electrolyte containing Cu^2+^. Micrometer-size Cu columns were obtained by retracting the microelectrode with a feedback loop maintaining a constant current of electrolysis. Microstructures of copper were also deposited from a solution of its stable complexes by using the SECM chemical lens concept.^75^ The electrolyte beneath the SECM tip is locally acidified by electrogeneration of protons. This causes local decomposition of complexes, and thus facilitates electroless or galvanic deposition of Cu at the surface below. Sarkar and Mandler employed SECM and SICM for indirect local deposition of Cu.^76^ First, Pd nanoparticles were locally deposited and used as the catalyst for Cu electroless deposition by immersing the Pd catalyst in the deposition bath. Various methods of manufacturing metal structures at the micrometer scale, which could be possibly applied to copper, are reviewed in ref. 77 and 78.

**Fig. 1:**
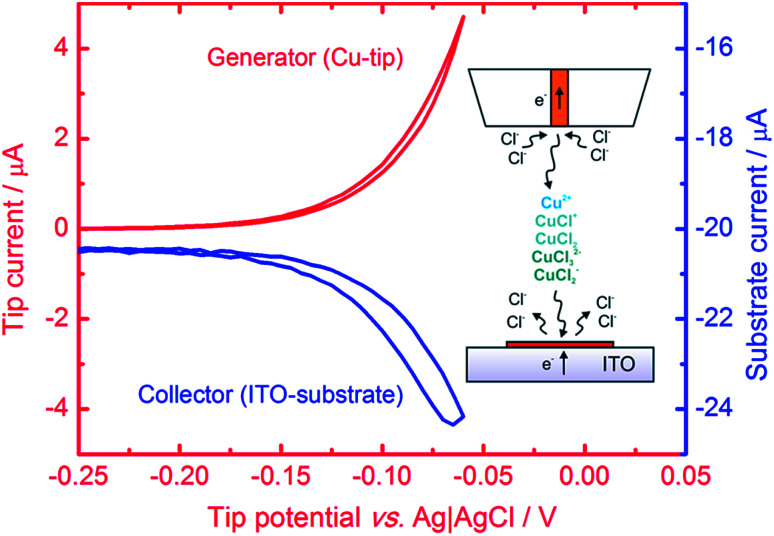
Cyclic voltammogram of a 100 μm diameter Cu electrode (tip, red line) positioned 30 μm above the ITO electrode (substrate) polarized at −0.5 V. The blue line is the substrate current *vs.* tip potential. Electrolyte: 1 M KCl + 10 mM HCl; scan rate: 50 mV s^−1^. The inset is a general scheme of localized electrorefining of Cu with SECM (not to scale).

Here we present a method of fabrication of bare (non-capped) copper nanostructures with a tailored morphology and catalytic activity towards the ORR in an alkaline environment and the CO_2_RR with the generation of compounds, which are electrochemically reoxidizable to CO_2_ at moderate anodic potentials. Micropatterns of CuNSs are obtained by localized electrorefining of polycrystalline Cu wire from a sacrificial microelectrode (Fig. 1, inset). Such an approach allows simple preparation of multiple patterns of nanostructures deposited on a single support sample under various conditions influencing the catalytic properties of the obtained nanomaterials. This enables rapid scanning electron microscopy (SEM) and microscale SECM analyses^79–82^ and optimization of the experimental parameters of the electrorefining process. The morphology and thus the electrocatalytic properties of CuNSs are tuned by adjusting the electrolyte composition, electrodeposition potential and Cu source translation rate. Although micrometer size model samples were fabricated and analyzed at the microscale, one can apply the methodology presented herein to larger scale fabrication of CuNSs.

## Experimental methods

### Chemicals

H_2_SO_4_, HCl, NaNO_3_ (Chempur), KCl (Sigma-Aldrich), NaOH (Fluka), and HCOOH (Sigma-Aldrich) were obtained. NaH_2_PO_4_ (Sigma) and Na_2_HPO_4_ (POCh) were used for aqueous electrolyte preparation with deionized water purified by using an Elix system (Millipore). Argon N5.0 was from Multax.

### Preparation of microelectrodes

Microelectrodes were fabricated according to a procedure described previously.^79^ Simply, 25 μm or 100 μm diameter wires of Pt (Mint of Poland), Au, or Cu (Alfa Aesar) were inserted in a borosilicate glass capillary. Then they were mounted into a PC-10 micropipette puller (Narishige) to melt the glass and to seal the wire tightly inside the capillary under vacuum conditions to avoid gas bubbles. The end of the microelectrode was polished with P2000 grit silicon carbide sand paper.

### Electrodeposition of Cu nanostructures

All electrochemical experiments were performed using a homemade scanning electrochemical microscope controlled and operated under SECMx software.^83^ The equipment consists of an Ivium CompactStat bipotentiostat and mechOnics XYZ positioning system, allowing positioning of the microelectrode (SECM tip) close to the sample – ITO coated glass (sheet resistance 8–12 Ω sq^−1^, Delta Technologies Ltd.) or a GC plate (Alfa Aesar). Ag|AgCl|3 M NaCl (ALS) and a Pt wire served as a reference and auxiliary electrode, respectively. All potentials in this report are provided *versus* the used reference. When the transparent ITO was used as the sample, the electrochemical cell was mounted on an inverted optical microscope (Nikon MA200) to see the progress of electrodeposition and to estimate the distance between the two working electrodes. For nontransparent GC, the distance between the electrodes was set by controlling the tip current corresponding to ORR. Electrodissolution of Cu microelectrodes was carried out at 0.15 V in 0.5 M H_2_SO_4_ or at −0.06 V in electrolytes containing chlorides. For SECM analysis of the obtained CuNSs the electrolyte was exchanged gradually under continuous cathodic polarization of the sample. Special attention was paid to prevent the electrodes (sample, reference and auxiliary) from losing their contact with the electrolyte.

### Sample characterization

In order to overcome the known problems caused by the instability of Cu,^19,76^ before the removal of CuNS samples from an SECM cell, an acidic electrolyte was exchanged gradually to 0.1 M phosphate buffer pH 7.2 under continuous cathodic polarization. The samples were rinsed with deionized water and dried with argon. The surface morphology and chemical composition of the deposited CuNSs were observed/examined with the use of a scanning electron microscope (SEM, FEI Nova NanoSEM 450 equipped with an EDAX energy dispersive X-ray spectroscopy (EDX) detector and GENESIS software). EDX analysis was performed at a primary beam energy of 20 kV.

## Results and discussion

### Morphology and composition of the deposited Cu nanostructures

Microsamples composed of CuNSs were prepared by localized electrorefining of a 100 μm diameter Cu microelectrode positioned 30 μm above the ITO support. The faradaic current recorded at the Cu source microelectrode upon its anodic polarization corresponds to electrodissolution of Cu (Fig. 1). The cathodically polarized ITO substrate (∼3.14 cm^2^) exhibits residual background current of a few tens of μA (depending on the applied potential) due to sluggish reduction of oxygen. Once electrodissolution of the Cu microelectrode starts, cathodic current at the ITO support starts to grow accordingly yielding under quiescent conditions (without Cu-tip movement) nearly 100% coulombic collection efficiency (see ESI S1†).

**Fig. 2:**
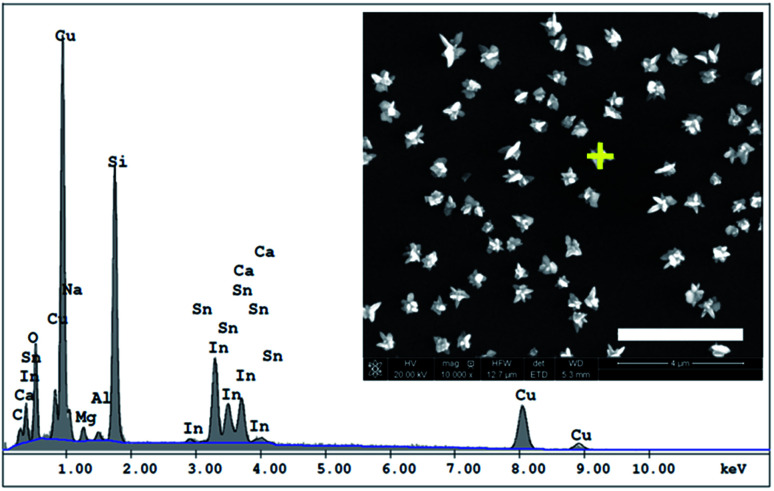
EDX spectrum of CuNSs deposited on the ITO coated glass support obtained by localized electrorefining under the following conditions – electrolyte: 1 M KCl + 10 mM HCl; electrodeposition potential: −0.5 V *vs.* Ag|AgCl; Cu source: 100 μm diameter Cu disk microelectrode; source translation velocity: 100 μm s^−1^. The inset provides the SEM image with the marked point of EDX analysis, scale bar: 4 μm.

The elemental composition of CuNSs on the ITO coated glass support was confirmed by EDX analysis (Fig. 2). The EDX spectrum shows the presence of copper in addition to other elements originating from the ITO (Sn, In, and O) coated glass substrate (Si, Al, Ca, and Mg).

**Fig. 3:**
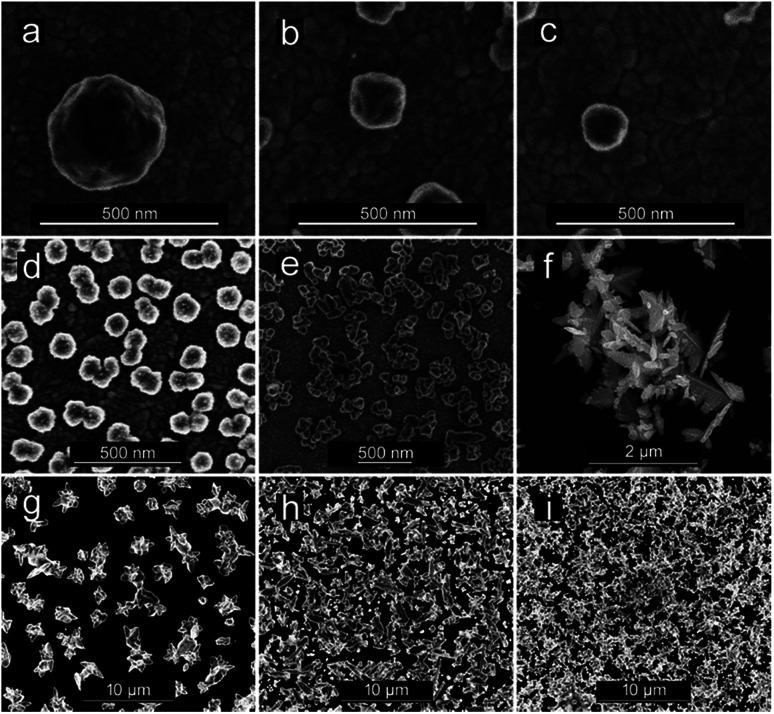
Copper nanostructures (CuNSs) obtained under the following conditions: (a–c) substrate electrode – ITO; electrolyte – 0.5 M H_2_SO_4_; substrate potential *vs.* Ag|AgCl: −0.2 V; microelectrode translation rates – 50 μm s^−1^ (a), 100 μm s^−1^ (b), and 200 μm s^−1^ (c). (d–f) Substrate electrode – ITO; substrate potential *vs.* Ag|AgCl: −0.6 V; microelectrode translation rate – 50 μm s^−1^; electrolytes – 0.5 M H_2_SO_4_ (d), 0.5 M H_2_SO_4_ + 0.1 M KCl (e), and 1 M KCl + 10 mM HCl (f). (g–i) Substrate electrode – glassy carbon; electrolyte – 1 M KCl + 10 mM HCl; microelectrode translation rate – 50 μm s^−1^; substrate potentials *vs.* Ag|AgCl: −0.3 V (g), −0.5 V (h), and −0.9 V (i).

CuNSs electrodeposited on ITO using 0.5 M aqueous H_2_SO_4_ as the electrolyte are always rounded, regardless of the potential value applied to the ITO substrate. Neither distinct edges nor sharp corners are seen in their SEM micrographs. The size of the obtained CuNSs depends on the translation rate of the electrodissolving Cu microelectrode. Faster horizontal translation rates of the Cu source result in smaller nanostructures (Fig. 3a–c). This is caused by the shorter time of exposure of a certain area of the ITO support to the Cu^2+^ flux evolving from the translating Cu microelectrode, whereas a stable electrodissolution rate is maintained (constant anodic current observed at the Cu microelectrode). When the electrodeposition potential is shifted to lower (more negative) values, the rounded shape of CuNSs is not affected; however, they are smaller and the number of objects per unit area increases (Fig. 3d).

Another factor influencing the morphology of CuNSs is the composition of the electrolyte. Addition of KCl to H_2_SO_4_ solution as well as complete substitution of H_2_SO_4_ in solution containing only Cl^−^ anions – 1 M KCl acidified with 10 mM HCl (in order to prevent hydrolysis of Cu^2+^ and to facilitate electrodissolution of Cu) – alters the shape of the CuNSs substantially (Fig. 3d–f). Cl^−^ at 1 M concentration causes the formation of stable CuCl^+^ and CuCl_2_ complexes.^84,85^ Moreover, Cu(i) complexes (CuCl_3_^2−^ and CuCl_2_^−^) are also stable under these experimental conditions, and thus electrorefining of Cu can occur with one electron per Cu atom stoichiometry. The amount of deposited copper estimated by analysis of SEM images is in good accordance with faradaic charge passed for the 2 electron reaction in H_2_SO_4_ solution and the 1 electron process in the presence of chlorides (see ESI S2†). The application of the chloride electrolyte decreases the electricity consumption required for electrorefining of Cu. This is especially important for large scale industrial processes. CuNSs obtained with the acidified KCl electrolyte are larger, less crowded on the ITO support and exhibit a number of sharp edges and corners (Fig. 3f). This is caused by the slower kinetics of nucleation and growth of Cu deposits from chloride-complexed copper ions. Negatively charged complexes are also repelled from the electrical double layer at the substrate upon its cathodic polarization (negatively charged substrate). Inhibited nucleation on the ITO surface causes larger separation of formed nuclei. Further growth of nanostructures causes depletion of copper ions around them preventing nucleation. The kinetics of outer sphere electroreduction of stable complexes depends on the type of crystallographic facet, and therefore electrodeposition on certain surface facets occurs faster than on others. This causes symmetry breaking and growth of prickly shaped CuNSs. Preferential adsorption of Cl^−^ on certain crystallographic facets can also play a role in the formation of prickly nanostructures. The influence of facet-dependent conductivity on chemical kinetics cannot be excluded.^86^ The increase of roughness of Cu deposits in the presence of Cl^−^ anions was recently reported by Suzuki *et al.*^87^ The electrodeposition of Cu on glassy carbon (GC) from a solution containing Cu–Cl complexes also produces CuNSs with numerous edges and corners (Fig. 3g). More negative electrodeposition potential applied to GC produces aggregates of smaller CuNSs. Although a similar amount of Cu is deposited, a larger surface is exposed with a possibly large number of low coordination Cu atoms at edges, corners and defects (Fig. 3h and i). Such a morphology is expected to be beneficial for electrocatalysis.

### CO_2_ reduction at Cu nanostructures

**Scheme 1:**
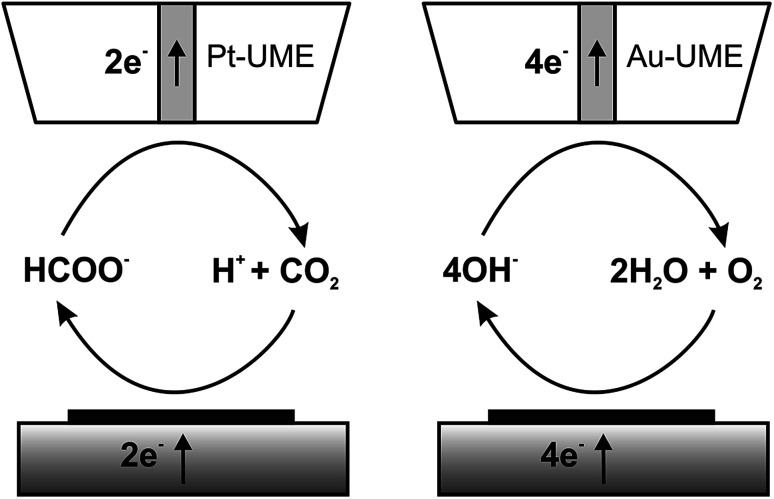
Schemes (not to scale) of processes occurring during feedback mode SECM analysis of the carbon dioxide reduction reaction (left) and the oxygen reduction reaction in alkaline media (right).

The catalytic properties of the electrodeposited nanostructures towards the CO_2_RR and ORR were studied using feedback mode SECM (Scheme 1). The feedback mode CO_2_RR was realized utilizing CO_2_ electrogeneration by oxidation of formic acid on a Pt electrode.^82^ CO_2_ from the Pt microelectrode (SECM tip) positioned above the CuNSs diffuses to the studied catalyst. At neutral pH it is reduced mainly to HCOOH,^33–35,82^ generating an additional flux of the tip reaction substrate. There are also other possible paths of the CO_2_RR yielding carbon monoxide, formaldehyde, methanol, ethanol, methane and ethylene.^29^ Contrary to other products, CO can be also reoxidized at the SECM tip to CO_2_ at moderate applied potentials. Continuous cycling of charge carriers (CO_2_ – dominating oxidized form; HCOOH – dominating reduced form) between the Pt tip and CuNS sample ensures feedback mode and allows testing of the catalytic properties of CuNSs by feedback current measurement at the tip. In neutral (pH 7) buffered solution hydrolyzed CO_2_ (p*K*_a1_(H_2_CO_3_) = 6.35)^88^ and HCOOH (p*K*_a_ = 3.75)^88^ occurs as bicarbonate (HCO_3_^−^) and formate (HCOO^−^), respectively. CuNSs deposited at ITO are not suitable for the CO_2_RR due to the instability of ITO under strong cathodic polarization,^89^ required to drive the CO_2_RR. Therefore, we employed glassy carbon as a support for electrodeposition of CuNSs for their further study as a CO_2_RR catalyst.

**Fig. 4:**
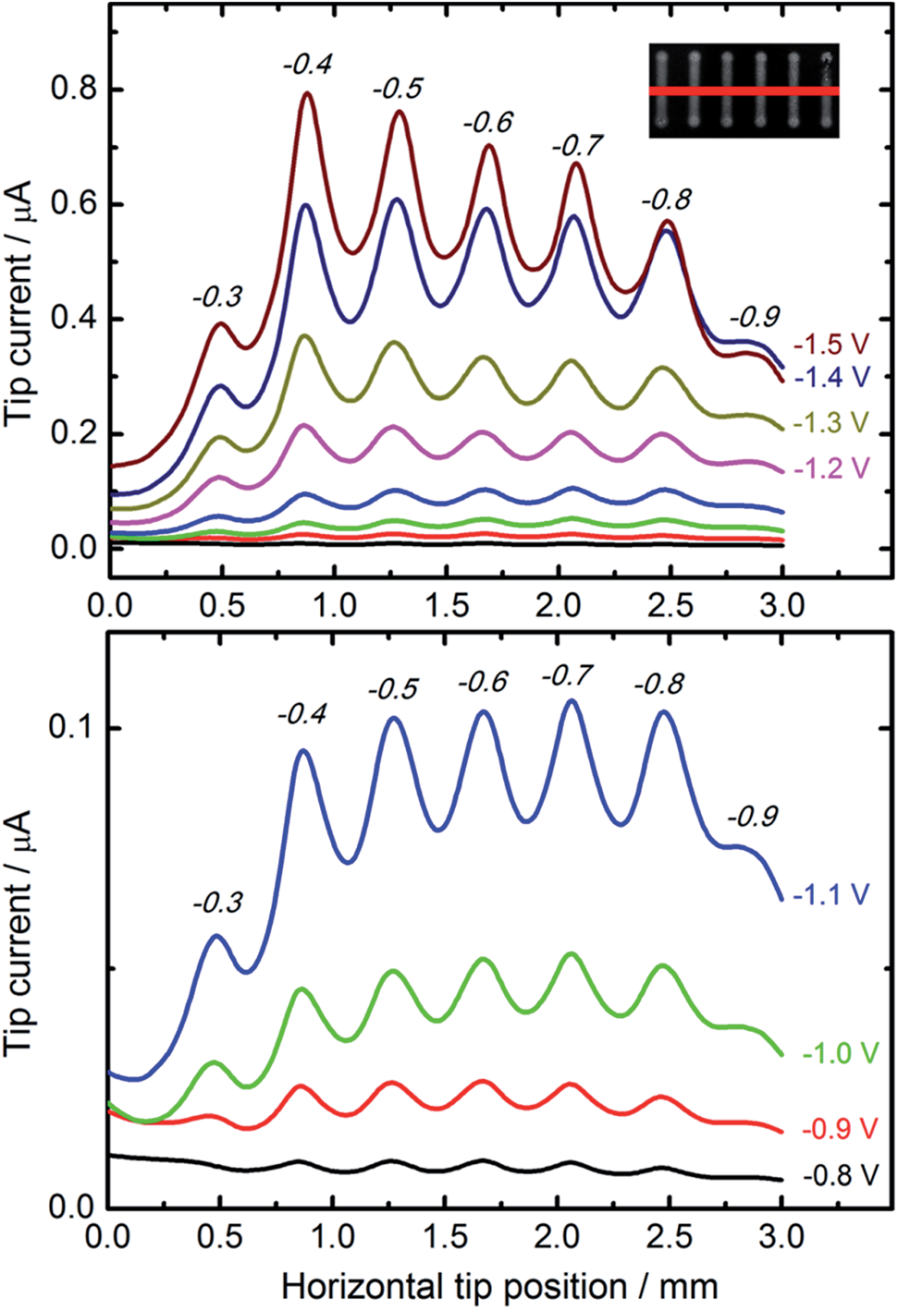
Feedback mode SECM line scan analysis result of the CO_2_RR occurring on the microstrips of CuNSs obtained by localized electrorefining at various substrate potentials. Electrodeposition potentials are marked above the current peaks (italicized, in volts). Other electrorefining parameters – substrate electrode: glassy carbon; Cu source microelectrode translation rate: 50 μm s^−1^; electrolyte: 10 mM HCl + 1 M KCl. In the analytical experiment a 100 μm diameter Pt microelectrode (tip) moved 30 μm above the sample in a direction perpendicular to the microbands (inset: red line) of CuNSs at 10 μm s^−1^. Tip potential: +0.7 V; sample potentials marked in the figure; reference electrode: Ag/AgCl; electrolyte: 30 mM HCOOH + 0.1 M phosphate buffer (pH 7).

For comparative studies of the catalytic properties of CuNSs towards the CO_2_RR we prepared parallel microstrips of nanostructures deposited at various potentials applied to a glassy carbon support. All the microstrips were prepared at the same source translation rate (50 μm s^−1^) to ensure an equal amount of deposited metal in each stripe. This sample was analyzed by SECM in a buffered solution of HCOOH with a Pt microelectrode tip. Fig. 4 shows horizontal line scans perpendicular to the axes of CuNS microstrips. Anodic feedback current recorded at the SECM tip is enhanced when scanning above cathodically polarized CuNSs compared to when the tip scans above the non-modified glassy carbon surface. Positive feedback current is due to catalytic regeneration of HCOOH on the electrodeposited CuNSs. CuNSs protrude only up to ∼1 μm above the flat support (see ESI S3†). Therefore the influence of sample topography on feedback current can be neglected (tip-to-sample distance: 30 μm). Since the CuNSs were prepared at different electrodeposition potentials a plot of tip current *vs.* horizontal tip position is used to compare the catalytic activities of these nanostructures. Clearly, there is a strong influence of electrodeposition potential on the electrocatalytic activity of CuNSs. For the CO_2_RR carried out at moderate potentials, down to −1.1 V *vs.* Ag|AgCl, the optimal value of electrodeposition potential is *ca.* −0.7 V. When the CO_2_RR is driven at more extreme cathodic polarization, CuNSs obtained at −0.4 V acquire maximum activity, instead. At potentials below −1.1 V applied to the CuNS sample, the hydrogen evolution reaction (HER, by electroreduction of water) may occur simultaneously with the CO_2_RR and contribute to the SECM feedback current. This result shows that the HER occurs faster at other types of active sites at CuNSs than the CO_2_RR. Because CuNSs deposited at more negative potentials possess more developed surface and less exposed flat crystallographic facets (see Fig. 3g–i), one could conclude that a chemically reversible CO_2_RR, contrary to the HER, occurs preferentially at low coordination sites (edges and corners) rather than at the crystallographic facets of CuNSs. This is in accordance with Ledezma-Yanez *et al.* observation that acetaldehyde reduction occurs at lower overpotentials at more open facets of Cu.^90^ Another reason for this behavior can be the larger increase of solution pH upon the CO_2_RR close to the CuNSs with a more developed structure due to a steric hindrance of mass transport of reactants near concave structures. Such an effect shifts the HER (1e^−^/1H^+^ stoichiometry) onset potential towards more negative values by 59 mV pH^−1^, contrary to ∼30 mV pH^−1^ in the case of the CO_2_RR (2e^−^/1H^+^ stoichiometry). Another reason for the larger SECM feedback current at CuNSs with a more developed structure can be related to CO_2_RR pathways.^29^ For HCOOH evolution and CO evolution, these products can be easily reoxidized to CO_2_ at a Pt microelectrode polarized at moderate anodic potentials. Other possible products of the CO_2_RR – HCHO, CH_3_OH, CH_4_, C_2_H_5_OH and C_2_H_4_ – despite their value as combustible fuels, are not as easily electrooxidizable as HCOOH and CO under applied experimental conditions (0.7 V *vs.* Ag|AgCl). Suppressed selectivity for multi-carbon compound formation upon the CO_2_RR on roughened Cu nanocubes was reported.^91^

SECM feedback mode screening of CO_2_RR catalysts provides valuable information about sample activity and chemical reversibility of catalyzed reaction. This is especially important when application of the CO_2_RR is considered in electrochemical energy storage (reversibility required). It is worth reminding that all the strips of CuNSs contain approximately the same amount of Cu per unit area of the support. This is due to the fact that at deposition potentials less than −0.2 V the CuNS growth rate is limited by diffusion. The same number of Cu ions is delivered per unit area of the substrate (constant parameters: electrodissolution current, tip translation rate, and tip-to-sample distance).

**Fig. 5:**
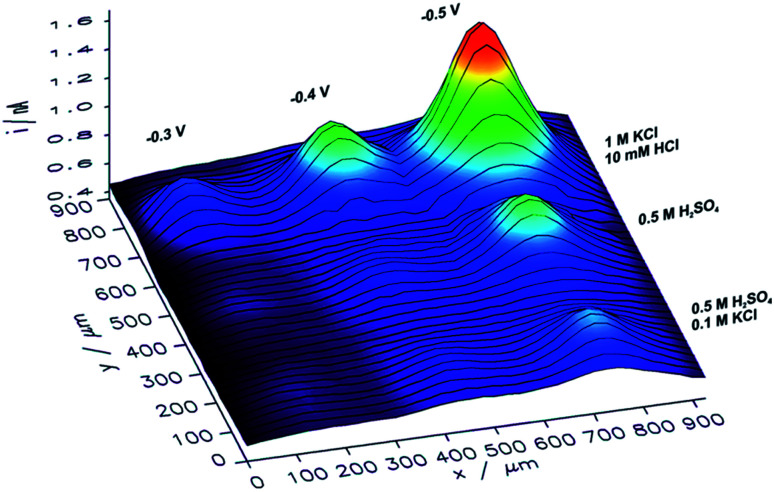
Feedback mode SECM image of the CO_2_RR at microspots of CuNSs deposited on a glassy carbon support at various deposition potentials applied to the support and with the use of various compositions of an electrorefining electrolyte (parameters marked in the figure). Sample potential during imaging: −0.9 V. Other imaging parameters are the same as in Fig. 4.

In order to analyze the influence of the electrolyte composition applied for electrorefining of copper on the catalytic properties of the obtained CuNSs, SECM mapping of CuNSs prepared at the GC electrode with three different solution compositions was performed. Localized electrorefining under quiescent conditions, *i.e.* without translating of the Cu microelectrode, was performed in the following electrolytes: (1) 0.5 M H_2_SO_4_, (2) 0.5 M H_2_SO_4_ + 0.1 M KCl, and (3) 1 M KCl + 10 mM HCl, at three different potentials applied to the GC (−0.3 V, −0.4 V, and −0.5 V). A map of SECM feedback current recorded in a similar way as described above is presented in Fig. 5.

CuNSs obtained in electrolytes containing only Cl^−^ anions (without SO_4_^2−^) exhibit the highest catalytic activity towards the CO_2_RR. CuNSs obtained with 0.5 M H_2_SO_4_ and H_2_SO_4_ with an addition of 0.1 M KCl exhibit similar activities substantially lower than those obtained with acidified electrolytes containing only chloride anions. Thus, optimization of the electrolyte composition should be focused on towards providing a high concentration of chloride and avoidance of sulphates. When stable Cu–Cl negatively charged complexes are formed, which require higher cathodic polarization for nucleation and growth of CuNSs, then CuNSs with exposed crystallographic facets are deposited (see Fig. 3). Such structures are preferred as catalysts for the CO_2_RR at high overpotentials, when the HER is also possible. In order to prevent the hydrolysis of Cu^+^/Cu^2+^ ions and precipitation of Cu(OH)_2_ pH has to be kept around 2 (10 mM HCl). Further pH decrease shifts hydrogen evolution potential to higher values affecting the electrodeposition of CuNSs. The largest activity towards the CO_2_RR was obtained for CuNSs deposited at −0.5 V. This electrodeposition potential is lower than that in the case of electrorefining of Cu with the microelectrode translating horizontally at 50 μm s^−1^ (Fig. 4). This shows that the mechanism of electrodeposition of CuNSs is significantly affected by hydrodynamic conditions caused by electrode motion. Indeed the morphology of CuNSs obtained under quiescent conditions differs from that obtained with the translating microelectrode (see ESI S4†). The most active CuNSs, obtained at −0.5 V applied to the GC support, are the smallest among those obtained under quiescent conditions. Their exposed surface area is the largest per amount of Cu deposited. Contrary to the spherical CuNSs obtained without chloride ions in the electrolyte, crystallographic planes are visible at their surfaces. This result confirms that the CO_2_RR with the generation of electrooxidizable compounds and possible contribution of the HER under strong cathodic polarization, besides spots with low coordination surface atoms, occurs also at flat crystallographic planes.

### Oxygen reduction at Cu nanostructures

**Fig. 6:**
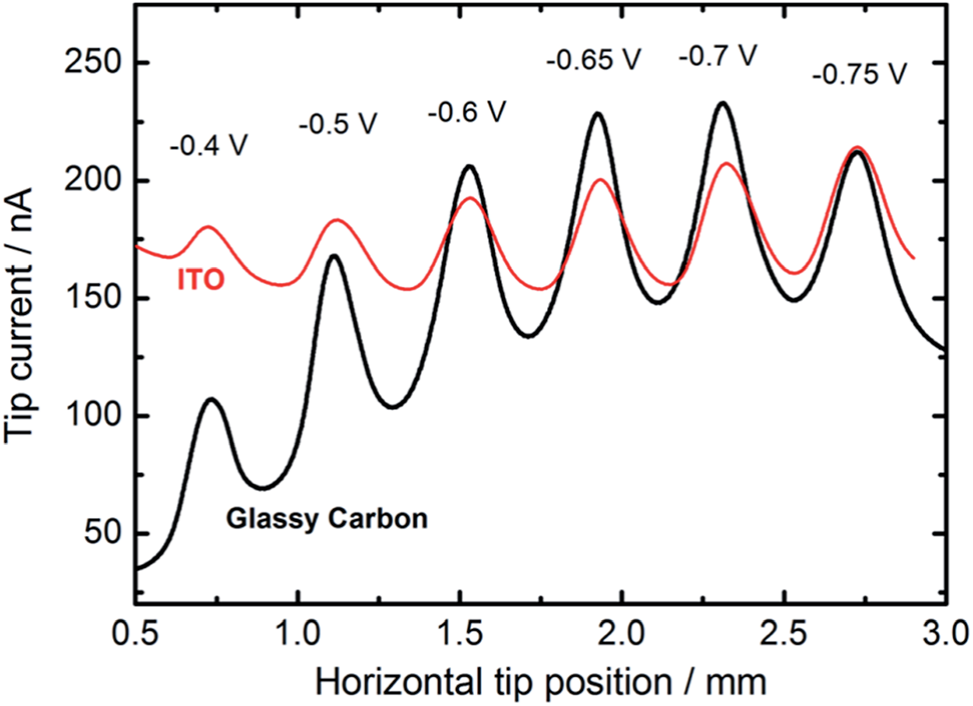
Feedback mode SECM line scan analysis result of the ORR occurring on the microstrips of CuNSs obtained by localized electrorefining at various substrate potentials and materials (marked in the figure). Other electrorefining parameters are the same as in Fig. 5. In the analytical experiment a 100 μm diameter Au microelectrode (tip) was translated 30 μm above the sample perpendicular to the micro-bands (see the inset in Fig. 5) of CuNSs at 10 μm s^−1^. Tip potential: +1.6 V; sample potential: −0.7 V; reference electrode: Ag/AgCl; electrolyte: 0.1 M NaNO_3_ + 10 mM NaOH.

We also employed feedback mode SECM to study the catalytic activity of CuNSs towards the ORR in alkaline solution.^92^ O_2_ is generated at the SECM tip (Au microelectrode) by OH^−^ oxidation^93^ (Scheme 1) and diffuses into CuNSs where it is re-reduced to OH^−^, generating an additional flux of OH^−^ anions enabling SECM feedback at the Au tip. Since H_2_O_2_ does not undergo oxidation at the Au microelectrode, the SECM positive feedback reveals only a 4-electron ORR at the examined CuNSs. Due to the less negative onset potential of the ORR as compared to the CO_2_RR, we were able to apply ITO as the support for electrodeposition of catalytic CuNSs and to compare the effect of the support (GC *vs.* ITO). Fig. 6 shows the results of SECM line scan analysis of the catalytic activity of microstrips of CuNSs towards the ORR. Microstrips were deposited on GC or ITO and analyzed under the same conditions. One can see slightly higher feedback currents for the CuNSs deposited on GC and maximal activity for CuNSs deposited at different potentials; however, the influence of the support material on the resulting activity of CuNSs is not significant. Feedback currents are at a similar level, but the contrast is higher for the sample deposited on the GC support. Lower current contrast for the ITO supported sample is due to the higher residual activity of ITO towards the ORR than the residual activity of GC. The most active CuNSs towards the ORR were prepared on glassy carbon at −0.7 V applied to the sample during electrorefining. This corresponds to the same conditions as in the case of the CO_2_RR under moderate polarization, when the HER is not involved. Therefore, a similar methodology can be applied for the preparation of CuNSs for both the CO_2_RR and the ORR. The same strategies of catalyst preparation do not apply to the HER.

**Fig. 7:**
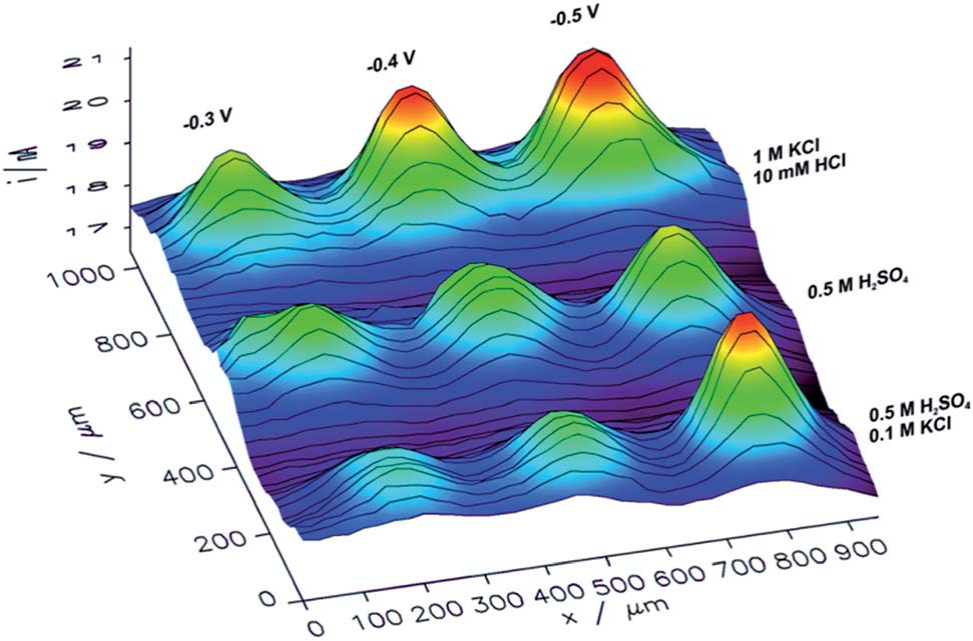
Feedback mode SECM image of the ORR at microspots of CuNSs deposited on an ITO support at various deposition potentials applied to the support and with the use of various electrolytes (parameters marked in the figure). Parameters of imaging are the same as in the Fig. 6 caption.

With an ITO substrate CuNSs deposited at −0.75 V exhibit the highest activity towards the ORR. One can suspect that electrodeposition at more negative potentials could yield CuNSs that are even more active. However, ITO electrodes are not suitable for polarization at lower potentials.^89^ Similarly to the CO_2_RR, the ORR occurs more efficiently at CuNSs obtained with the use of an electrolyte containing a high concentration of chloride ions (Fig. 7). This approach facilitates symmetry breaking and formation of spiky-shaped crystalline CuNSs, which both for the CO_2_RR and ORR are more active than amorphous structures obtained without chloride ligands.

## Conclusions

We have presented a simple methodology of preparation of bare (non-capped) copper nanostructures from a polycrystalline metallic source. This procedure relies on localized electrorefining of copper microwires constituting the tip of a scanning electrochemical microscope. The size and morphology of the obtained nanostructures and thus their catalytic properties can be tuned by adjusting the electrorefining parameters, *i.e.*, the applied voltage, the source translation rate and the composition of the supporting electrolyte. Scanning electrochemical microscopy analysis of microarrays of nanostructures allows their quick characterization and optimization as catalysts for the CO_2_RR to electrooxidizable fuels useful for storage of renewable energy. Electrorefining of copper with the use of chloride ions at a high concentration results in prickly nanostructures exhibiting the highest catalytic activity. Structures deposited under high cathodic overpotentials possess a high surface-to-volume ratio with a large number of catalytic sites active towards the carbon dioxide reduction process yielding easily electrooxidizable compounds. The oxygen reduction reaction in alkaline media occurs effectively at the same sites, whereas the hydrogen evolution reaction occurring simultaneously with the CO_2_RR at high cathodic overpotentials is probably catalyzed rather at the crystallographic facets of larger copper nanostructures electrodeposited under moderate overpotentials. Despite the catalytic properties of bare Cu nanostructures, their non-capped surface is prone to corrosion and adsorptive contamination resulting in activity deterioration. In the case of electrocatalysis long-term activity can be provided by periodic depassivation and electrodesorption enforced by polarization of the electrode within the available potential window.

## Conflicts of interest

There are no conflicts to declare.

## Supplementary Material

NA-001-C9NA00166B-s001
