# Supplementary material for: Patterning Cu nanostructures tailored for CO2 reduction to electrooxidizable fuels and oxygen reduction in alkaline media
Source: Nanoscale Adv. 2019 May 20;1(7):2645–53. doi: 10.1039/c9na00166b (PMC9416923; doi:10.1039/c9na00166b)
Supplement: NA-001-C9NA00166B-s001 [file NA-001-C9NA00166B-s001.pdf]

Electronic Supplementary Material (ESI) for Nanoscale Advances.
This journal is © The Royal Society of Chemistry 2019

Supplementary information

Patterning Cu nanostructures tailored for CO_2 reduction to electrooxidizable fuels and oxygen reduction in alkaline media

Magdalena Michalak, Agata Roguska, Wojciech Nogala * and Marcin Opallo *

Institute of Physical Chemistry, Polish Academy of Sciences, Kasprzaka 44/52, 01-224 Warsaw, Poland

*E-mail: wnogala@ichf.edu.pl (WN), mopallo@ichf.edu.pl (MO)

S1. Estimation of collection efficiency of electrorefining of copper

Figure S1 shows processes data from Figure 1 in the paper. Substrate current (cathodic) modulus is corrected by subtraction of constant background to get zero values for potential range, where no electrodissolution of copper occurs. Although tip current reaches higher values at -0.06 V, the moduli of charges are very similar. This is due to the time delay of electrodeposition at the substrate versus electrodissolution at the tip due to diffusion of generated copper ions. Slightly higher cathodic charge recorded at the sample is most probably caused by electroreduction of oxygen on freshly prepared Cu nanostructures (CuNS). This result suggest nearly 100% collection efficiency under quiescent conditions, *i.e.* without tip movement.

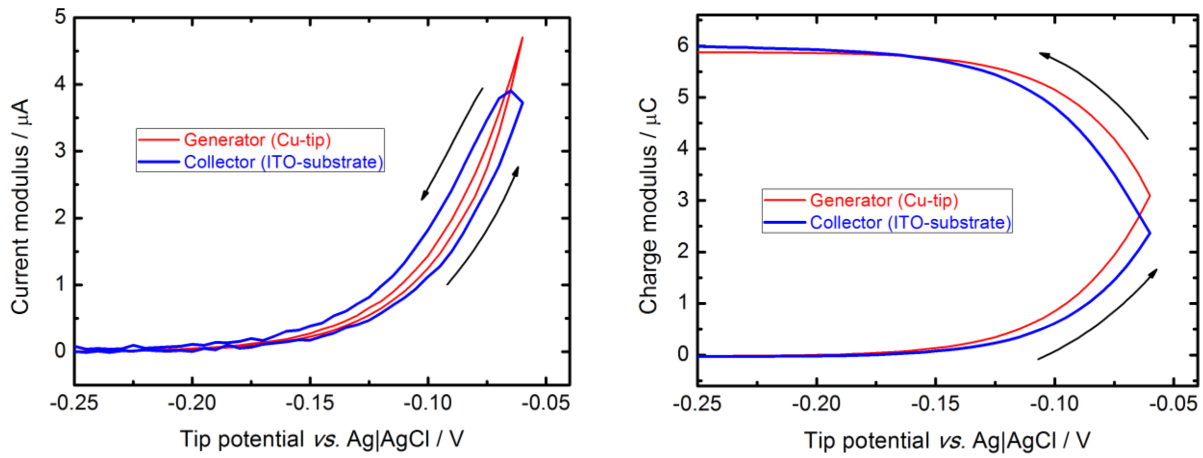

Figure S1. Left plot – Cyclic voltammogram of a 100 µm diameter Cu electrode (tip, red line) positioned 30 µm above the ITO electrode (substrate) polarized at -0.5 V. The blue line is the substrate current corrected by subtraction of constant background. Electrolyte: 1 M KCl + 10 mM HCl; scan rate: 50 mV s^{-1}. Right plot – Cyclic voltammograms from the left plot integrated over time to get charge-potential plots.

S2. Estimation of surface concentration of electrodeposited copper

In order to estimate size distribution of CuNS selected gray-scale SEM images were converted to 1-bit monochrome ones with a threshold adjusted to get black projections of CuNS on a white background. The separated nanostructures were counted and the projected area of each spot was calculated by pixel area integration (Figure S2). In order to minimize the particles size estimation errors, their projected areas (regions surrounded by black pixels in the mask image) were separated by intuitive manual cutting in the graphics software. The projected areas of each particle were evaluated using the ImageJ free software. The 'diameters' ($d = (4A/\pi)^{1/2}$) and volumes (Equation S1) of CuNS were estimated under the assumption of spherical shape:

$$V = \frac{4A^{3/2}}{3\pi^{1/2}} \quad \text{(S1)}$$

where *A* is the projected area of the nanostructure.

The sum of volumes of all the particles visible in the SEM image divided by the image area (5.56 µm^2) and multiplied by the volumetric mass density of Cu (8.95 g/cm^3) provides the surface concentration of electrorefined metal. This method of volume estimation is accurate exclusively for ideal spheres.

For example, the volume of hemisphere is half of the value calculated by Equation S1. Side view SEM images CuNS (Figure S3) reveal that nanostructures are grown rather perpendicularly to the ITO surface. This is in accordance with precursor concentration gradient normal to the substrate surface upon electrorefining. Application of Equation S1 to assess the volume of CuNS on the basis of their projected area should therefore result in volume values being close to actual volumes.

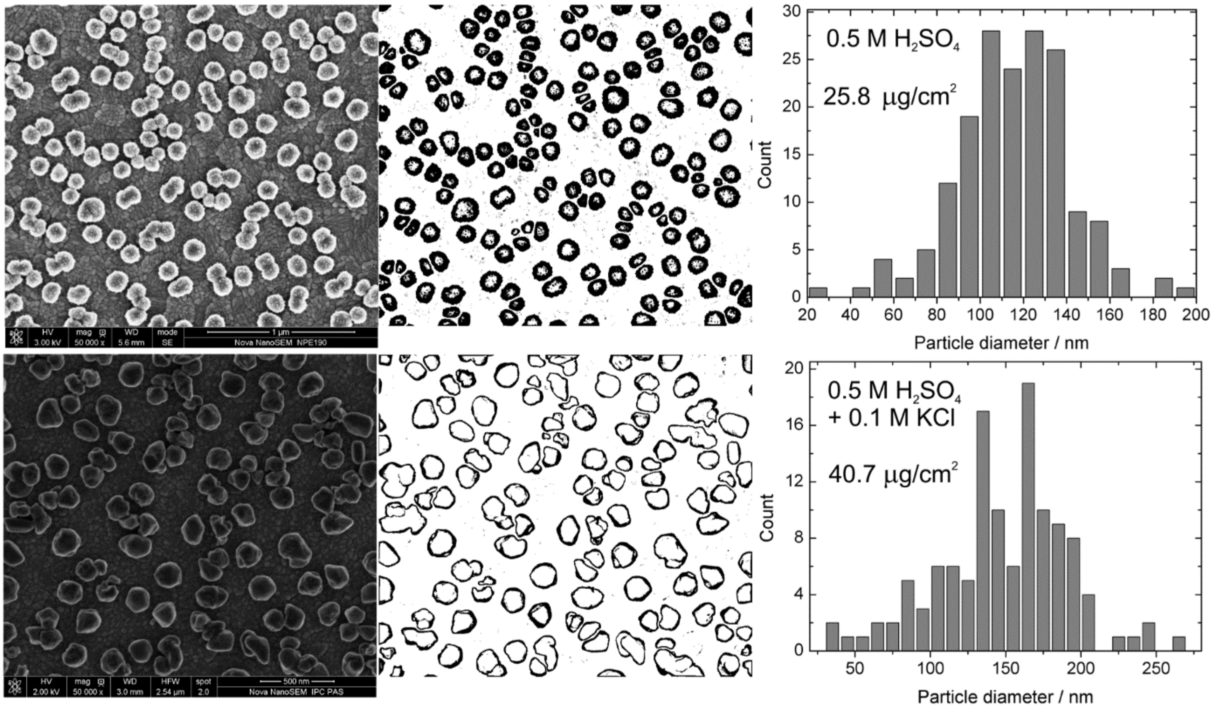

Figure S2. Nanostructure size estimation by microscopic analysis. Raw SEM images (left column) calibrated to real distance was converted to particles mask (center column). CuNS size distribution histograms (right column) estimated as hypothetical spheres with the same projected area. CuNS were obtained by electrorefining of a 100 µm diameter Cu microelectrode translating horizontally 30 µm above the ITO electrode polarized at -0.4 V *vs.* Ag|AgCl. Microelectrode translation rate: 50 µm/s. Electrolytes: 0.5 M H_2SO_4 (upper row), 0.5 M H_2SO_4 + 0.1 M KCl (bottom row).

Under an assumption that the majority of Cu is deposited beneath the tip source, one can calculate theoretical surface concentration of metal in deposited micro-bands using the Faraday laws of electrolysis and simple geometry (Eqation S2):

$$\Gamma = \frac{i_T \theta}{zF\phi_T v} \qquad \text{(S2)}$$

where Γ is the surface concentration (mol cm^{-2}), i_T is the tip current (A), θ is the collection efficiency (0.8 for v = 50 µm s^{-1}),[S1] z is the number of electrons transferred per atom (or ion), F is the Faraday constant (96485 C mol^{-1}), ϕ is the tip diameter (cm), and v is the tip translation velocity (cm s^{-1}).

The parameters of deposited CuNS are summarized in Table S1. The surface concentration of Cu obtained with electrolyte without chloride anions, estimated by analysis of SEM images (25.8 µg cm^{-2}) is in good accordance with the value calculated using equation S2 and two-electron stoichiometry (24 µg cm^{-2}). Analysis of SEM image of CuNS deposited with electrolyte containing 0.1 M KCl results in the amount of Cu (40.7 µg cm^{-2}), which is closer to the theoretic value calculated for one-electron stoichiometry (47.8 µg cm^{-2}). This shows that addition of chloride ions at concentration as low as 0.1 M stabilizes Cu(I) complexes ($CuCl_3^{2-}$ and $CuCl_2^{-}$) facilitating 1-electron electrorefining.

Table S1. Geometric parameters of CuNS obtained by analysis of SEM images and Faradaic charge.

Electrolyte	Particle diameter (nm)	Γ_{SEM} (µg cm^{-2})	i_T (µA)	z	$\Gamma_{Faradaic}$ (µg cm^{-2})
**0.5 M H_2SO_4**	115 ±26	25.8	4.55	2	24
**0.5 M H_2SO_4 + 0.1 M KCl**	148 ±42	40.7	4.54	1	47.8

S3. Side view SEM micrographs of CuNS

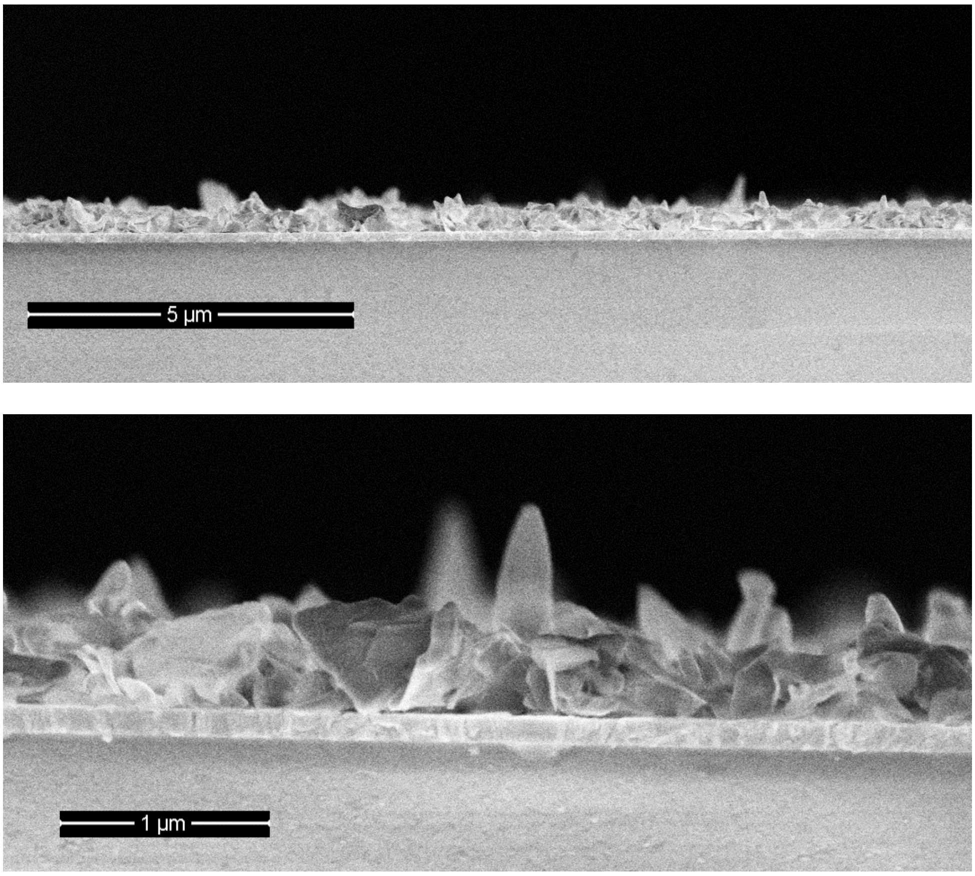

Figure S3. SEM micrographs of CuNS obtained by localized electrorefining under the following conditions; Supporting material: ITO; Electrolyte: 1 M KCl + 10 mM HCl; Electrodeposition potential: -0.5 V *vs.* Ag|AgCl; Cu source: 100 µm diameter Cu disk microelectrode; Source translation velocity: 50 µm s^{-1}. The sample was broken manually to allow side view SEM imaging.

S4. SEM images of CuNS deposited under quiescent conditions

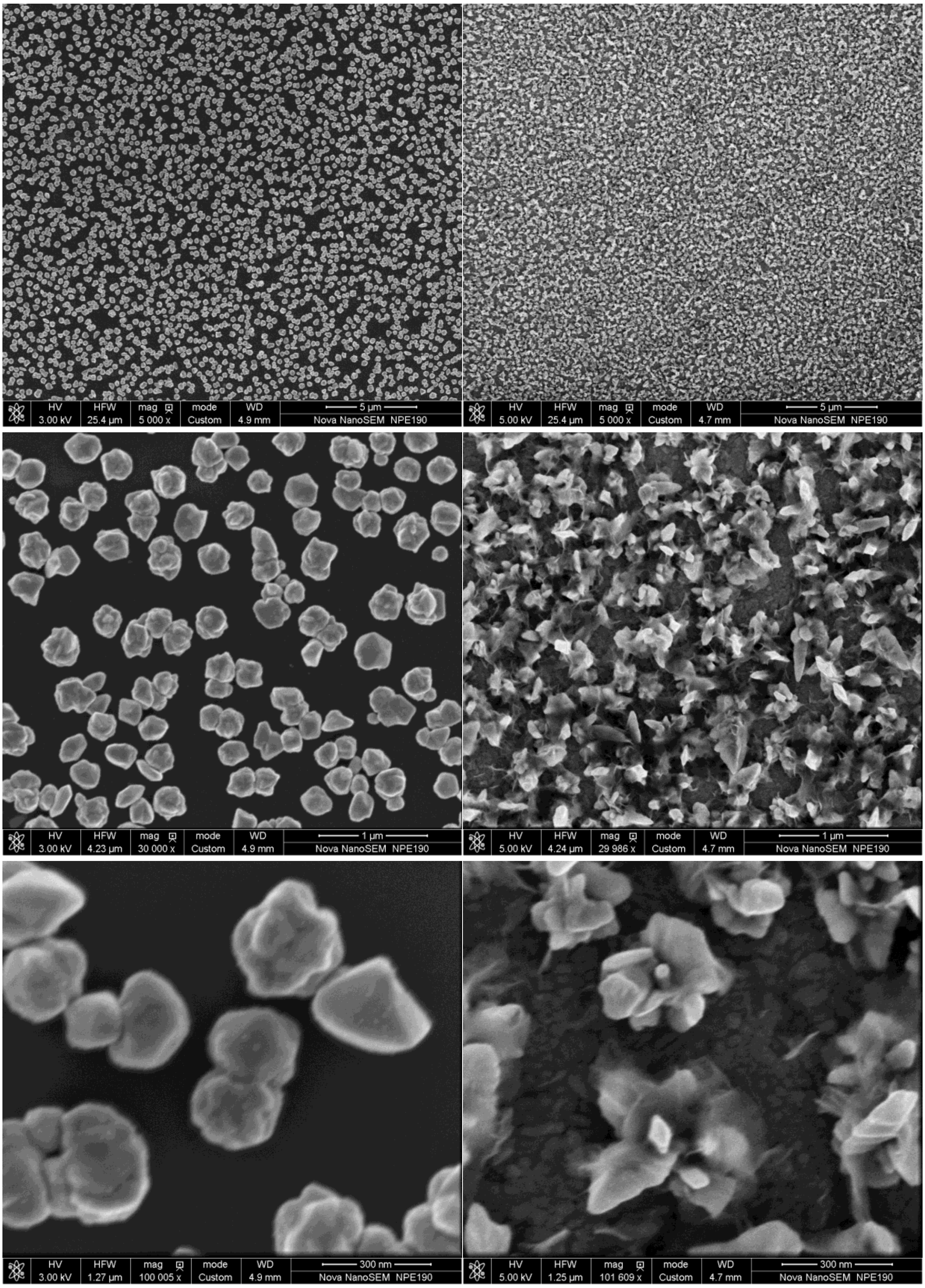

Figure S4. SEM micrographs (various magnifications) of CuNS obtained by localized electrorefining under the following conditions; Supporting material: glassy carbon (left column), ITO (right column); Electrolyte: 1 M KCl + 10 mM HCl; Electrodeposition potential: -0.5 V *vs.* Ag|AgCl; Cu source: 100 µm diameter Cu disk microelectrode; Distance: 30 µm; Source potential pulse: -0.06 V *vs.* Ag|AgCl for 2 seconds.

References

S1. W. Nogala, P. Kannan, S. Gawinkowski, M. Jönsson-Niedziolka, M. Kominiak, J. Waluk and M. Opallo, *Nanoscale*, 2015, **7**, 10767–10774.
